# ACE gene polymorphisms (rs4340) II and DI are more responsive to the ergogenic effect of caffeine than DD on aerobic power, heart rate, and perceived exertion in a homogeneous Brazilian group of adolescent athletes

**DOI:** 10.1590/1414-431X2024e13217

**Published:** 2024-06-17

**Authors:** H. Spineli, M. dos Santos, D. Almeida, D. Gitaí, M. Silva-Cavalcante, P. Balikian, T. Ataide-Silva, A. Marinho, F. Sousa, G. de Araujo

**Affiliations:** 1 Instituto de Educação Física e Esporte, Universidade Federal de Alagoas, Maceió, AL, Brasil; 2 Faculdade de Nutrição, Universidade Federal de Alagoas, Maceió, AL, Brasil; 3 Instituto Federal de Educação Ciência e Tecnologia de Alagoas, Maceió, AL, Brasil; 4 Instituto de Ciências Biológicas e da Saúde, Universidade Federal de Alagoas, Maceió, AL, Brasil

**Keywords:** Genetic polymorphism, Teenagers, Angiotensin, Physical performance, Ergogenic effects

## Abstract

The purpose of this study was to verify the association between angiotensin-converting enzyme (ACE) genotypes DD, DI, and II and caffeine (CAF) ingestion on endurance performance, heart rate, ratio of perceived exertion (RPE), and habitual caffeine intake (HCI) of adolescent athletes. Seventy-four male adolescent athletes (age: DD=16±1.7; DI=16±2.0; II=15±1.7 years) ingested CAF (6 mg/kg) or placebo (PLA) one hour before performing the Yo-Yo Intermittent Recovery level 1 (Yo-Yo IR1) test. No difference was found among groups for HCI. However, CAF increased the maximal distance covered and VO_2_max in DI and II genotype carriers compared to PLA (DD: Δ=31 m and 0.3 mL·kg^-1^·min^-1^; DI: Δ=286 m and 1.1 mL·kg^-1^·min^-1^; II: Δ=160 m and 1.4 mL·kg^-1^·min^-1^). Heart rate of DI and II genotype carriers increased with CAF compared to PLA, while RPE was higher in the II and lower in the DD genotypes. The correlations between HCI and maximal distance covered or VO_2_max were significant in the II genotype carriers with CAF. CAF increased endurance capacity, heart rate, and RPE in adolescent athletes with allele I, while endurance performance and aerobic power had a positive correlation to HCI in the II genotype group. These findings suggested that DD genotype were less responsive to CAF and that genetic variations should be taken into account when using CAF supplementation to enhance exercise performance.

## Introduction

It is well known that caffeine supplementation has a positive effect on exercise performance (1,2). Its ergogenic effects are more frequently reported in endurance tasks (3,4), while they remain controversial in high-intensity and short-duration efforts, particularly strength-power performance (5,6). Caffeine acts primarily on central sites as an antagonist of adenosine receptors, leading to increased neural excitability (7). This in turn enhances muscle recruitment (8) and reduces the perception of exertion during exercise (9). In skeletal muscle, caffeine reduces potassium concentrations in plasma (10) and enhances muscle contractility and oxygen saturation (11). Furthermore, caffeine increases ventilation and heart rate response, resulting in higher maximal oxygen uptake (VO_2_max) (12). These physiological mechanisms help to construct a paradigm to explain the effects of caffeine in a broad range of exercises, especially of long duration.

Despite the several physiological mechanisms that may influence exercise tolerance in both high-intensity, shorter-duration exercises and longer-duration exercises, some effects of caffeine are restricted to the latter. In skeletal muscle, caffeine has been shown to increase the sensitivity of free-fatty acid (FFA) oxidation specifically in type I fibers compared to type II fibers during *in vitro* contraction (13). This finding may explain the greater impact of caffeine on endurance exercises (14,15). Even though the ergogenic effect of caffeine is overall well accepted for endurance tasks, its positive responses are not always present when analyzing individual data (16,17). Based on this framework, it is worth noticing that carriers of the I allele in the ACE rs4340 polymorphism, especially II homozygotes, are more predisposed to aerobic endurance performance due to predominance of type I fibers (18). These type I fibers are more suited for aerobic activities due to their oxidative capacity. However, it is still unknown whether carriers of the I allele are more responsive to acute caffeine supplementation.

Inter-individual variability in response to caffeine has been attributed to genetic polymorphism (19). Several studies have explored the link between caffeine and CYP1A2 and ADORA2A (20), which are considered key genes to the ergogenicity of caffeine during endurance tasks. However, other polymorphisms may also contribute to individual responses during endurance tasks. For example, the presence of the D allele for ACE polymorphism is associated with greater activation of the angiotensin II growth factor, deactivation of the bradykinin growth inhibition factor, higher muscle strength and power, and predominance of type II muscle fibers (21). On the other hand, the association of the D allele with caffeine (CAF) supplementation has not been investigated yet. Similarly, the insertion (I) ACE polymorphism (287bp-intron 16) is more associated with endurance activities due to predominance of type I skeletal muscle fibers (22), as supported by muscle biopsy (18) and higher VO_2_max (23) than the deletion (D) allele. Previous studies investigating the impact of genetic variation on sports performance have been conducted in various populations (20). Due to the distinctive genomic characteristics of the mixed Brazilian population and differences in the proportion of ancestry compared to other populations, the interpretation, transferability, and interchangeability of genetic data can be challenging (24). Consequently, studies are needed regarding the association between ACE genetic variations and their effects on endurance performance, heart rate, perceived exertion ratio, and habitual caffeine intake (HCI) in Brazilian athletes.

Although the ergogenic effects of caffeine are less explored in adolescents (16,25), studies suggest an effect similar to adults (26,27). For example, moderate to high doses of caffeine (approximately 100-400 mg) led to increased reports of nervousness and tremors and decreased reports of sluggishness in children and adolescents (26,27). Perhaps, the short-term phenotypic exposure and recommendation of more controlled caffeine consumption made this sample more homogeneous and sensitive to treatment compared to adults.

The purpose of the present study was to investigate the effect of acute caffeine ingestion on aerobic performance, heart rate, ratio of perceived effort, and HCI in adolescent athletes with different ACE genotypes. We hypothesized that acute CAF responsiveness to endurance performance, heart rate, and perceived effort is influenced by the presence of the I allele of the ACE polymorphism (287bp-intron 16).

## Material and Methods

### Participants

Seventy-five adolescent male athletes who regularly trained at least three times a week participated in this study. The athletes were participants in sports such as volleyball, track and field, and soccer. Participant's characterization is displayed in Table 1. Participants and their parents were informed about the experimental risks and signed a consent form before starting the experiments. The study procedures were conducted in accordance with the Declaration of Helsinki (2008) and were approved by the Federal University of Alagoas Ethics committee (number 1.541.599).

**Table 1:** Anthropometric characteristics of study participants.

	DD (n=22)	DI (n=40)	II (n=13)
Age (years)	16±1.7	16±2.0	15±1.7
Height (cm)	170±8	169±1	167±1
Body mass (kg)	62.4±9.4	59.7±12.3	51.1±9.1
BMI (kg/m^2^)	21.4±2.4	20.5±2.5	18.1±1.6

The data are reported as means±SD. BMI: Body mass index.

### Experimental protocol

The study was conducted using a randomized, crossover, and double-blind design. Participants visited the laboratory three times with a minimum of a 72-h interval. In the first visit, a caffeine consumption questionnaire, anthropometric measurements, blood collection, and familiarization with all procedures adopted in the experimental trials were performed.

The subjects were weighed on an electronic scale with 0.1 kg precision (Supermedy, Brasil). Height was measured using a measuring tape placed on the wall. Blood collection was performed by peripheral phlebotomy in four-mL vacuum tubes with EDTA (BD Vacutainer^®^, USA). The tubes were stored at -20°C until DNA extraction.

To assess HCI, a caffeine consumption frequency questionnaire was applied. Participants indicated the amount and frequency of each food on the list. To calculate daily CAF consumption, it was assumed that 150 mL of pure coffee contained 100 mg of CAF; 28 g milk chocolate, 6 mg CAF; 250 mL of energy drink, 80 mg of CAF; 350 mL of cola, 46 mg of CAF; 150 mL of tea, 30 mg of CAF; 150 mL of coffee with milk, 33 mg of CAF; and 350 mL of guarana soda, 2 mg of CAF (28).

On the second and third visits, a capsule containing 6 mg/kg of anhydrous caffeine (CAF) or cellulose (PLA) with a similar size, weight, and color was administered with 200 mL of water one hour before the tests. The participants performed a battery of short-duration tests (in sequence: handgrip strength, agility, push-ups, countermovement jumps, spike jumps, sit-ups) followed by the Yo-Yo Intermittent Recovery test level 1 (Yo-Yo IR1) (28,29). Participants were instructed to maintain their regular eating habits and avoid exhaustive exercise, caffeine, alcohol, or nutritional supplements 24 h prior experimental trials.

### Yo-Yo IR1

Following the protocol by Bangsbo et al. (30), the subjects performed laps (20+20 m), accompanied by a sound signal at the start, middle, and end of the race, with a brief recovery period of 10 s after every 40 m. The velocity increment was controlled at the end of each stage. The test starts with four bouts between 10 and 13 km/h (0-160 m), seven bouts of 13.5-14 km/h (160-440 m), followed by stages of eight shuttle runs with an increment of 0.5 km/h. The tests were conducted until the subject's exhaustion, characterized by missing the signals three consecutive times or by voluntary exhaustion. The VO_2_max was calculated by the formula: VO_2_max (mL·kg^-1^·min^-1^) = IR1 distance (m) × 0.0084 + 36.4.

### Genotyping

#### DNA extraction

All genotyping was performed by a blinded investigator not involved with the experimental protocol. DNA extraction from 300 μL of blood was performed by using FlexiGene DNA Kit (Qiagen, USA), according to the recommendation of the supplier.

#### DI ACE, rs4340

The analysis of the ACE polymorphism was performed as described by Gómez-Gallego et al. (31). The two alleles of the human ACE gene were identified by the insertion or deletion of a 287 bp repeat element in intron 16. The PCR conditions were as follows: initial denaturation at 95°C for 5 min; 35 cycles at 95°C for 30 s, 57°C for 30 s, 72°C for 1 min, and a final extension at 72°C for 5 min. The fragments were detected on a 1.5% agarose gel stained in ethidium bromide. To avoid misclassification, a second PCR was performed on all samples classified as DD with the specific primer pairs: 5'-TGGGACCACAGCGCCCGCCACTAC-3' (forward) and 5'-TCGCCAGCCCTCCCATGCCCATAA-3' (reverse). The PCR conditions were similar to those described above, except for the annealing temperature (63°C). Only the I allele produces a 335 bp fragment, identified on 2.0% agarose gel stained with ethidium bromide. The ACE DI fragments with deletion (D allele) and with insertion (I allele) were identified as 190 and 490 bp, respectively. The DI genotype has the two forms of fragments.

### Patient and public involvement

Patients have been involved in the development and refinement of the experimental protocol, and the authorship team includes three subjects who were managed with the methods.

### Equity, diversity, and inclusion

The participants were a cohort of mostly black and indigenous adolescents. The region of the state of Alagoas is characterized primarily by a black and indigenous population. This region has the worst human development index of the country. The sports program in which the participants take part is essential for social inclusion, talent detection, and bringing adolescents closer to university.

### Statistical analyses

The data are reported as means±SD. Statistical analyses were performed using a statistical package (Statistica^®^ version 10.0, StataSoft and Social Science Statistics^®^, USA). Data distribution was checked by the Kolmogorov-Smirnov test. Once normal distribution was confirmed, one-way ANOVA for independent groups was used to compare the HCI among genotypes. Student's *t*-test for 2 dependent means was used for intra-genotype groups comparisons. Pearson correlation test was used to calculate the correlation between caffeine intake (mg/day), VO_2_max, and total distance covered (m) for DD, DI, and II genotypes in both conditions (PLA and CAF). Cohen's effect size (ES- Θ) was also calculated for differences in endurance performance where thresholds for small, moderate, and large effects were set at 0.20, 0.50, and 0.80, respectively. The level of significance was set at P≤0.05.

## Results

### HCI (mg/day)

**Figure 1:**
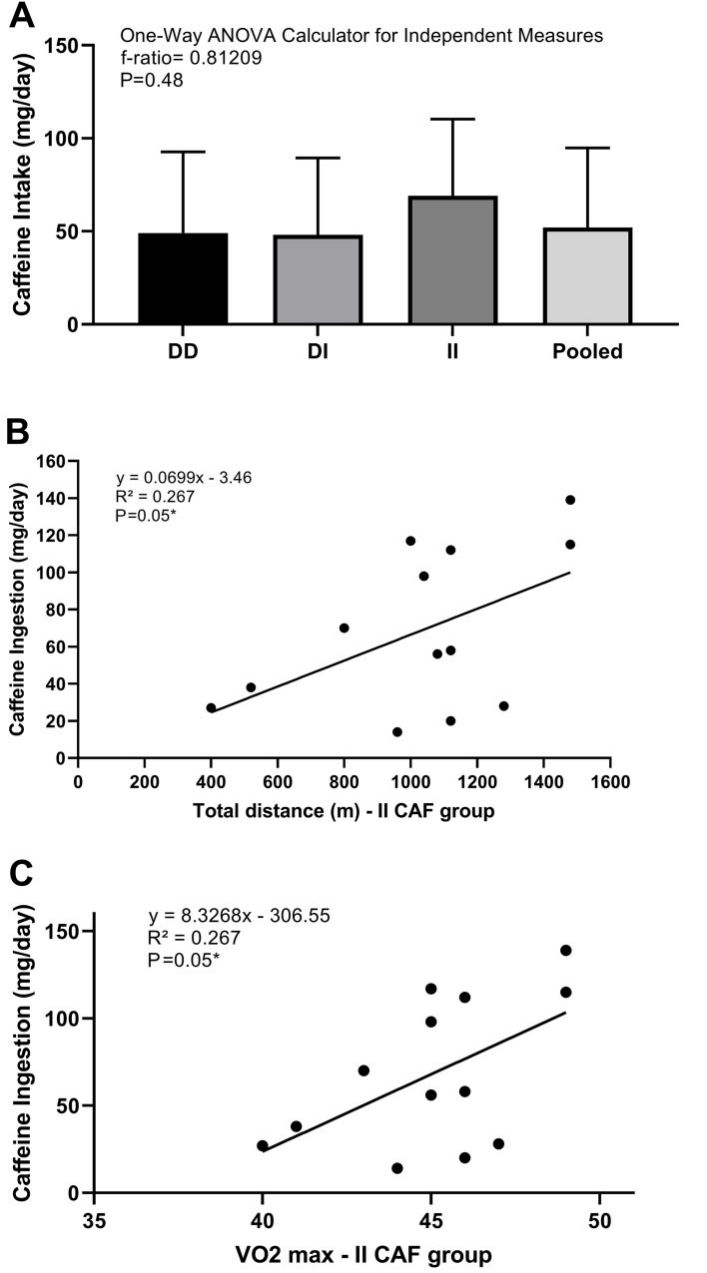
Habitual caffeine consumption for DD (n=22), DI (n=40), and II (n=13) angiotensin converting enzyme (ACE) polymorphism groups (**A**), Pearson correlation between caffeine ingestion and total distance covered (m) for the carriers of II in the caffeine (CAF) group (**B**), and caffeine ingestion and VO_2_max (mL·kg^-1^·min^-1^) for the carriers of II in the CAF group (**C**). The data are reported as means±SD.

HCI according to each group of the ACE polymorphism is described in Figure 1A. No differences were found among groups. There was a moderate effect size between II *vs* DI (effect size Θ=0.5) and II *vs* DD (effect size Θ=0.5). The effect size among other groups was considered small.

### Total distance (m) and VO_2_max (mL·kg^-1^·min^-1^)

CAF supplementation in the ACE DI and ACE II groups increased the total distance covered and VO_2_max in the Yo-Yo IR1 group compared to PLA (Table 2).

**Table 2:** Comparison between effects of placebo (PLA) and caffeine (CAF) intake in different groups of ACE genetic polymorphisms in the performance of the Yo-Yo IR1 test for total distance covered, VO_2_max, heart rate, and ratio of perceived exertion (RPE).

	Total distance (m) ACE DD	Total distance (m) ACE DI	Total distance (m) ACE II
PLA	956±340	914±315	871±303
CAF	987±357	1200±404	1031±305
*t*-value	0.636	3101.002	2053.343
P-value	0.26	0.001*	0.03*
	VO_2_max (mL·kg^-1^·min^-1^) ACE DD	VO_2_max (mL·kg^-1^·min^-1^) ACE DI	VO_2_max (mL·kg^-1^·min^-1^) ACE II
PLA	44.4±2.9	44.1±2.6	43.7±2.5
CAF	44.7±3.00	45.2±3.4	45.1±2.6
*t*-value	0.334575	3068.591	2501.064
P-value	0.37	0.001*	0.01*
	Heart rate (bpm) ACE DD	Heart rate (bpm) ACE DI	Heart rate (bpm) ACE II
PLA	189±13	193±14	195±9
CAF	189±9	198±8	199±10
*t*-value	-0.014778	2257.767	2317.731
P-value	0.49	0.01*	0.02*
	RPE (6-20) ACE DD	RPE (6-20) ACE DI	RPE (6-20) ACE II
PLA	18±3	16±3	17±2
CAF	17±3	17±3	20±3
*t*-value	2175.213	121.621	1924.654
P-value	0.01*	0.11	0.03*

The data are reported as means±SD. *P<0.05 between PLA and CAF. ACE: angiotensin converting enzyme.

### Heart rate and RPE

CAF treatment in the ACE DI and ACE II groups increased the heart rate (bpm) and RPE only for ACE II after Yo-Yo IR1 compared to PLA (Table 2).

### Correlations

There was a significant correlation between HCI (mg/day) and total distance (m) for ACE II in CAF condition (P=0.05, Figure 1B), but not for PLA (P=0.90). No significant correlations were found in ACE DI participants who took PLA and CAF (P=0.36; P=0.85), as well as ACE DD for PLA (P=0.69) and CAF (P=0.76). For VO_2_max, a positive correlation with HCI (mg/day) was found for ACE II in CAF condition (P=0.05, Figure 1C), but not for PLA (p=0.87). No significant correlations were found in ACE DI in both conditions, PLA and CAF (P=0.39; P=0.79), as well as ACE DD for PLA (P=0.69) and CAF (P=0.86), respectively.

### Frequency distribution for total distance (m)

The frequency distribution for ACE II for delta (Δ) between CAF and PLA demonstrated that 84.6% of group had a positive response of supplementation. For ACE DI group, 77.5% was positively sensitive to CAF administration. The frequency distribution for ACE DD reduced the CAF responsiveness for 66.7% of the participants (Table 3).

**Table 3:** Caffeine responsiveness evaluated from delta (Δ) distance (m) between caffeine (CAF) and placebo (PLA) supplementation and the frequency distribution for II, DI, and DD angiotensin converting enzyme (ACE) polymorphisms.

Class	Count	Percentage
Frequency distribution-ACE II-Δ Distance (m)		
-499	2	15.4
0-499	10	76.9
500-999	1	7.7
Total	13	100
Frequency distribution-ACE DI-Δ Distance (m)		
-400-201	5	12.5
-200-1	4	10.0
0-199	12	30.0
200-399	15	37.5
400-599	2	5.0
600-799	2	5.0
Total	40	100
Frequency distribution-ACE DD-Δ Distance (m)		
-500-251	3	14.3
-250-1	4	19.0
0-249	13	61.9
250-499	1	4.8
Total	21	100

## Discussion

To the best of our knowledge, this study was the first to investigate the association of ACE polymorphisms and CAF consumption and supplementation on physical performance in adolescent athletes. Our results confirmed the hypothesis that carriers of the ACE gene polymorphisms (rs4340) II and DI were more responsive to the ergogenic effect of CAF ingestion than DD carriers on aerobic power, heart rate, and perceived exertion. Furthermore, the endurance capacity was positively correlated to HCI only for the II group.

**Figure 2:**
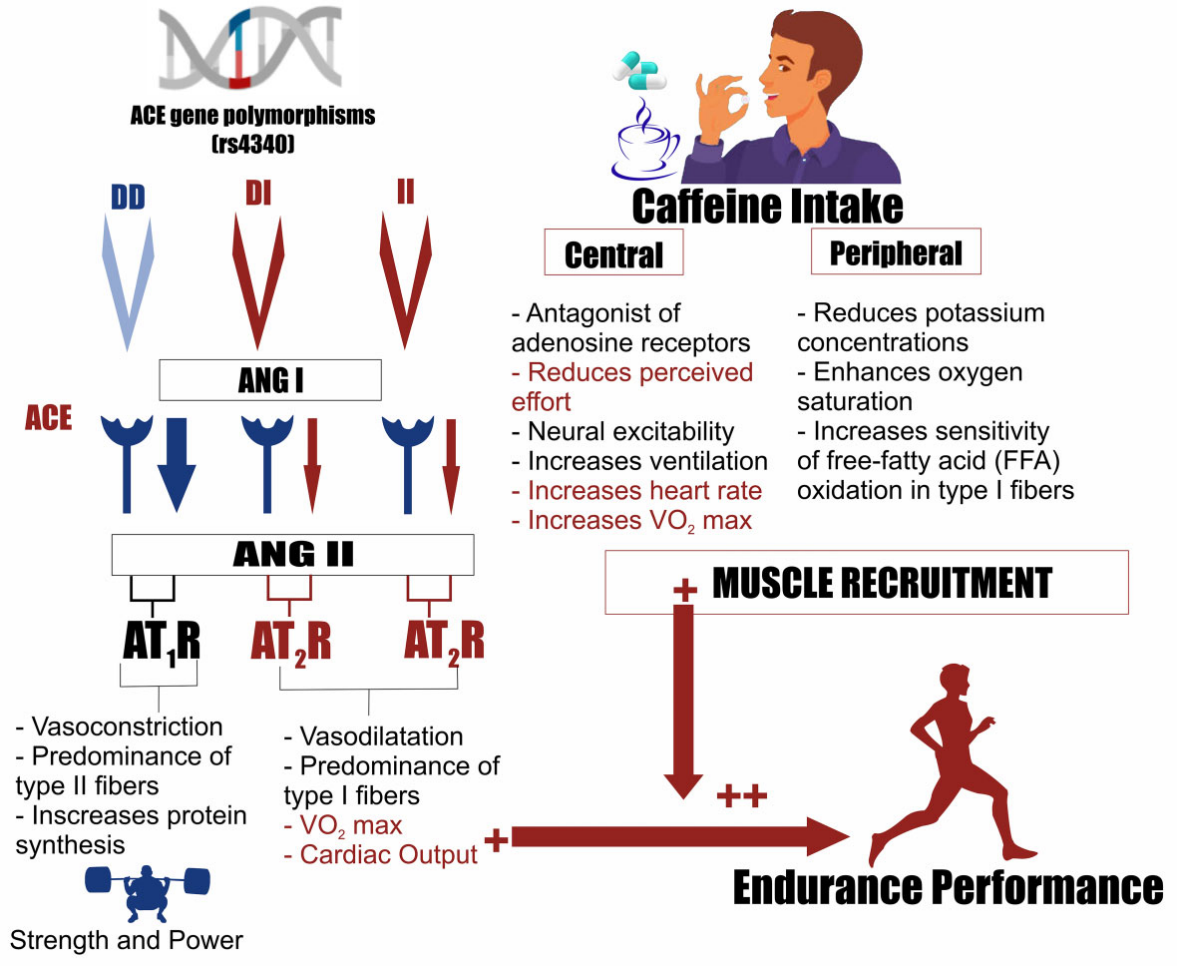
Schematic representation created with BioRender.com of the genetic contribution of angiotensin converting enzyme (ACE) gene polymorphism and mechanisms of action of caffeine intake. The information in red is a hypothetical synergic mechanism between genetic polymorphisms and caffeine intake to improve endurance exercise performance based on our data.

Evidence of the independent effects of ACE polymorphism as well as caffeine supplementation on aerobic performance is well consolidated (for a detailed summary of mechanism related to ACE gene polymorphism and caffeine intake, see Figure 2). However, no studies regarding the combined effects of CAF supplementation and ACE polymorphisms on endurance performance have been carried out.

Athletes carrying the ACE gene polymorphisms II and DI had improved performance in endurance exercise after caffeine ingestion. On the other hand, carriers of the ACE gene polymorphism DD had lower ergogenic effects of caffeine. Although no previous studies regarding specific II and DI gene polymorphism are available, the result was not in line with previous studies with adolescents (28,29). In these studies, caffeine intake improves endurance, regardless of the gene polymorphism (28,29). The difference between our study and the previous study may be attributed to the gene polymorphism assessed. Some authors assessed interactions between CYP1A2 (28) and ADORA2A (29) gene polymorphisms and caffeine intake, which are only reported for caffeine metabolism. In the present study, the ACE gene polymorphisms were assessed, which are related to important morphological and physiological alterations (e.g., typology of muscle fibers) regarding endurance exercise performance, and thus more susceptible to the ergogenic effects of caffeine. The distinct responses observed between carriers (II and DI) and non-carriers (DD) of the I allele regarding endurance exercise performance may be attributed to morphological predisposition of these participants. The carriers of allele I in the ACE polymorphism are more directed to endurance performance due to the predominance of type I fibers (18), while the D allele carriers have a predominance of type II muscle fibers, which are associated with high muscle strength and power (21). These morphological differences may explain specific gains on endurance exercise performance in both the II and DI ACE gene polymorphisms.

Improvements in endurance after caffeine intake, as observed in the present study, have been well-documented in a previous study (32). The explanation regarding these benefits may be associated with the mechanisms of action of caffeine (32). The main mechanism of action of caffeine on the central nervous system is antagonist to that of adenosine (33), maintaining high neuronal excitability during exercise (7). In the peripheral regions, caffeine may improve the contractile function of skeletal muscles (34) to meet the physical demands of exercise, especially endurance performance.

Improved endurance of carriers of II ACE gene polymorphisms was accompanied by higher heart rate and RPE. Caffeine effects on heart rate (35) and RPE (9) have been well-discussed previously. As the II carriers maintained a longer exercise time in the caffeine condition than in the placebo condition, a higher degree of internal load, such as higher heart rate and RPE values, was expected. Elevated heart rate values with caffeine may be attributed to higher levels of adrenaline released during exercise (36). Caffeine facilitates adrenaline release to maintain neuronal excitability and a high skeletal muscle activity during exercise; however, longer exercise time that is accompanied by adrenaline release may also result in an enhanced heart rate response, once adrenaline binds to the adrenergic receptors in the heart, and thus, increases its beating rate (37). RPE is generally lower after caffeine intake, because of the delayed perception of effort exertion (9). However, in the current study, the variables were assessed at the moment of exhaustion, being higher for the CAF group. This may be explained by the longer distance covered during the Yo-Yo IR1, possibly causing a high degree of metabolic disturbances, thus influencing the RPE response. RPE is also modulated via III and IV afferent nerves during exercise (38), altering the perceived effort. Therefore, the longer exercise time after caffeine intake may have contributed to the increase in the systemic and perceptual responses.

Another interesting and the first reported result was the moderate association between endurance performance and HCI only for the carriers of II ACE gene polymorphisms. Previous studies showed associations between HCI and genetics (39,40). However, this association it not well understood. Although HCI seems to slightly modulate exercise performance (4), this response may not be a determinant for performance improvements.

### Limitations

Three main limitations of the present study must be acknowledged: 1) the test to evaluate the VO_2_max was indirect, based on ventilation, O_2_, CO_2_ kinetics, and others. Thus, further studies should measure VO_2_max by a direct test; 2) despite the large sample size (n=74), the rarity of homozygous II genotypes in the population limited the number of participants for the main group of the experimental response; 3) the study lacked physiological, metabolic, and molecular analyses to explain the possible interactions between the mechanisms of action of caffeine and the ACE polymorphisms.

### Conclusion

In conclusion, 6 mg/kg of CAF increased endurance capacity, heart rate, and perceived exertion in adolescent athletes that were ACE I allele carriers, but not in ACE DD genotype. Furthermore, endurance performance was associated with habitual caffeine consumption, specifically in the II genotype group.
